# The Neurexin superfamily of *Caenorhabditis elegans*

**DOI:** 10.1016/j.gep.2010.10.008

**Published:** 2010-11-03

**Authors:** Liat Haklai-Topper, Jürgen Soutschek, Helena Sabanay, Jochen Scheel, Oliver Hobert, Elior Peles

**Affiliations:** 1 Department of Molecular Cell Biology, The Weizmann Institute of Science, Rehovot 76100, Israel; 2 Department of Biology, Eberhard-Karl-Universität Tübingen, Germany; 3 Department of Biochemistry and Molecular Biophysics, Howard Hughes Medical Institute, Columbia University Medical Center, New York, NY 10032

**Keywords:** Neurexins, cell adhesion molecules, C.elegans

## Abstract

The neurexin superfamily is a group of transmembrane molecules mediating cell-cell contacts and generating specialized membranous domains in polarized epithelial and nerves cells. We describe here the domain organization and expression of the entire, core neurexin superfamily in the nematode *C. elegans*, which is composed of three family members. One of the superfamily members, *nrx-1*, is an ortholog of vertebrate neurexin, the other two, *itx-1* and *nlr-1*, are orthologs of the Caspr subfamily of neurexin-like genes. Based on reporter gene analysis, we find that *nrx-1* is exclusively expressed in most if not all cells of the nervous system and localizes to presynaptic specializations. *itx-1* and *nrx-1* reporter genes are expressed in non-overlapping patterns within and outside the nervous system. ITX-1 protein co-localizes with β-G-spectrin to a subapical domain within intestinal cells. These studies provide a starting point for further functional analysis of this family of proteins.

## 1. INTRODUCTION

**Fig.1:**
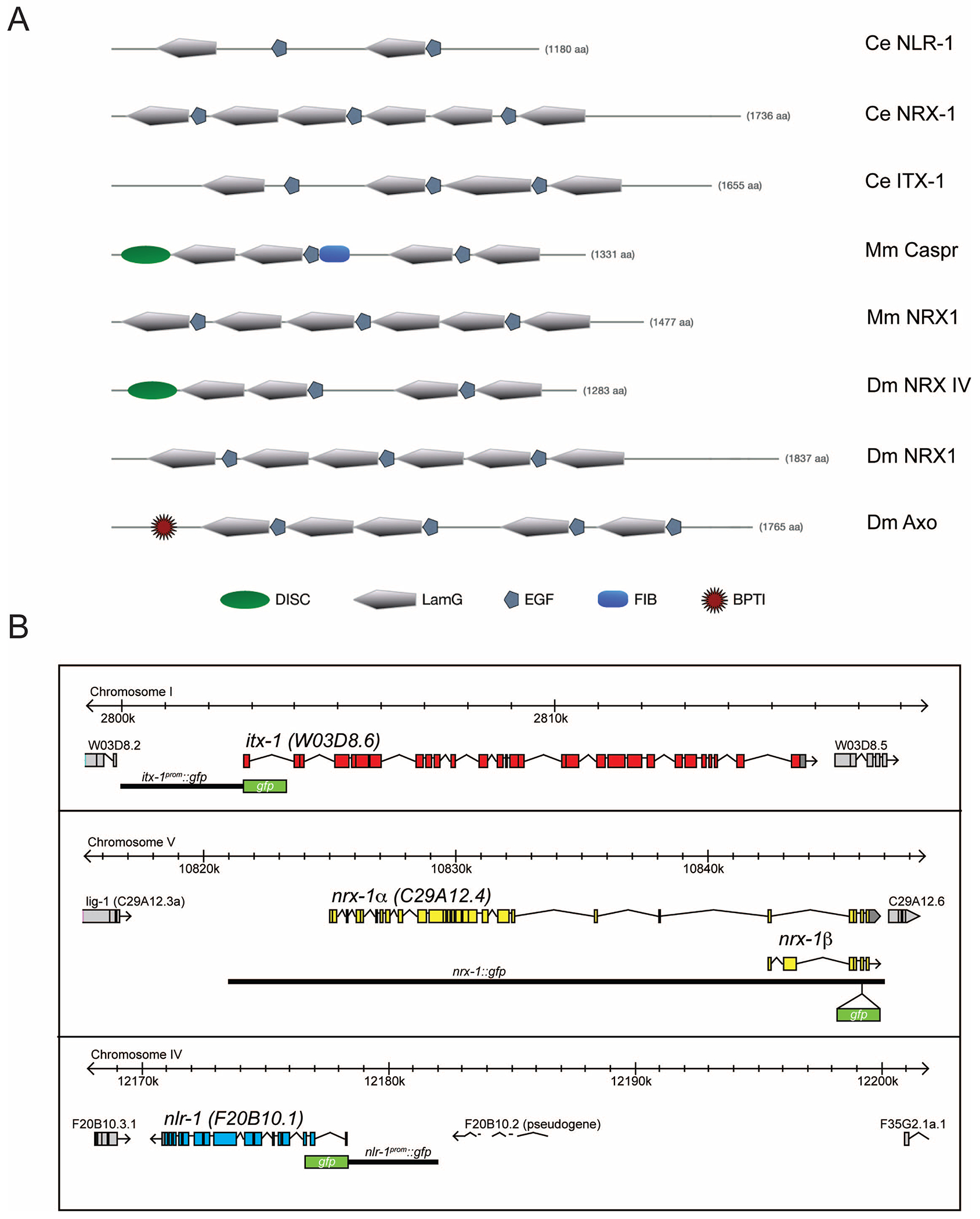
The neurexin superfamily in *C. elegans* **A:** Domain organization of neurexin superfamily members. NLR-Neurexin- like receptor 1; NRX-Neurexin; ITX-Intexin; AXO-Axotactin. DISC, discoidin I-like domain (also called F5/8 type C, or C1/C2- like domain); LamG, laminin globular (G) domain; EGF, EGF-like domain, FIB, Fibrinogen-like domain; BPTI (bovine pancreatic trypsin inhibitor) domain found in Kunitz family of serine protease inhibitors. **B:** Gene structure, as annotated in Wormbase. Transcript analysis of *nrx-1* demonstrated the existence of two splice forms, α and β. Structure of reporter gene constructs are indicated.

The neurexin superfamily of cell adhesion molecules contains two main subgroups, the classical neurexins and members of the Caspr subfamily (Lise and El-Husseini, 2006). In vertebrate, three genes encode the classical neurexins, each giving raise to two different transcripts (i.e., α and β forms) as a result of alternative promoter usages (Missler et al., 1998; Ushkaryov et al., 1992). In addition, the neurexins are subjected to extensive splicing, which result in the generation of a large number of multiple isoforms (Ullrich et al., 1995). The extracellular domain of α-neurexins contain three modular repeats that are composed of two laminin G domains that flank an EGF-like domain (Fig.1A). The extracellular domain of β-neurexins is short and contains a single laminin G domain. The Caspr proteins (for Contactin associated protein), which in humans are encoded by five distinct genes, also contain a discoidin and a fibrinogen-like domains in their extracellular region (Peles et al., 1997; Peles and Salzer, 2000)(Fig.1A).

Neurexins are trans-synaptic cell adhesion molecules that mediate neuron-neuron interaction and the functional organization of synapses (Lise and El-Husseini, 2006; Sudhof, 2008). Two of the five Caspr proteins (i.e., Caspr and Caspr2) mediate neuron-glia interaction and are essential for the functional organization of myelinated axons (Bhat et al., 2001; Boyle et al., 2001; Feinberg et al., 2010; Gollan et al., 2003; Poliak and Peles, 2003; Poliak et al., 2003; Traka et al., 2003). Mutations in genes coding for neurexin family members have been associated with a wide range of neuropsychiatric diseases (Sudhof, 2008)

With the exception of a single divergent member (*bam-2*), which is involved in axon branching (Colavita and Tessier-Lavigne, 2003), members of the neurexin superfamily have not yet been comprehensively described in the *Caenorhabditis elegans* model system. We provide here an overview of the number, structural organization and expression pattern of neurexin superfamily members in *C. elegans*.

## 2. RESULTS

### 2.1. Sequence analysis

Reciprocal BLAST searches demonstrate that the *C. elegans* genome contains four genes coding for members of the neurexin superfamily: the as yet uncharacterized *nrx-1, itx-1* and *nlr-1* core family members and the previously described, more divergent *bam-2* gene (Fig.1). All four genes code for transmembrane proteins with short cytoplasmic tails and laminin-G type domains and EGF domains in their extracellular region. Based on primary sequence identity, one protein, NRX-1 is most closely related to vertebrate neurexin proteins (Tabuchi and Sudhof, 2002), while the other two, ITX-1 and NLR-1, are orthologs of the CASPR-family of adhesion proteins (Fig.1A). ITX-1 and NLR-1 lack the N-terminal discoidin domain and the PGY repeats found in the fly and mammals CASPR orthologs. In addition, the C-terminal SH3 binding domain is replaced with a PDZ binding motif making these ITX-1 and NLR-1 more closely related to Caspr2 and Caspr3. Another protein in the *C. elegans* genome, BAM-2 (Colavita and Tessier-Lavigne, 2003) is a more divergent neurexin family member, with laminin-G and EGF-like domains that are not readily detectable by InterPro Scan analysis (Quevillon et al., 2005).

The gene structure of the 3 core members of the family is shown in Fig.1B. The *nlr-1* locus is unusual since a part of the 5’ end of the gene appears to have duplicated, with the duplicate probably constituting a pseudogene (www.wormbase.org). In striking similarity to vertebrate neurexins, the *nrx-1* locus produces a long and a short splice forms, α and β (Fig.1B). Additional splice forms have also been detected by RT-PCR analysis (J.S. and J.S., data not shown).

### 2.2. Reporter gene expression pattern

**Fig.2:**
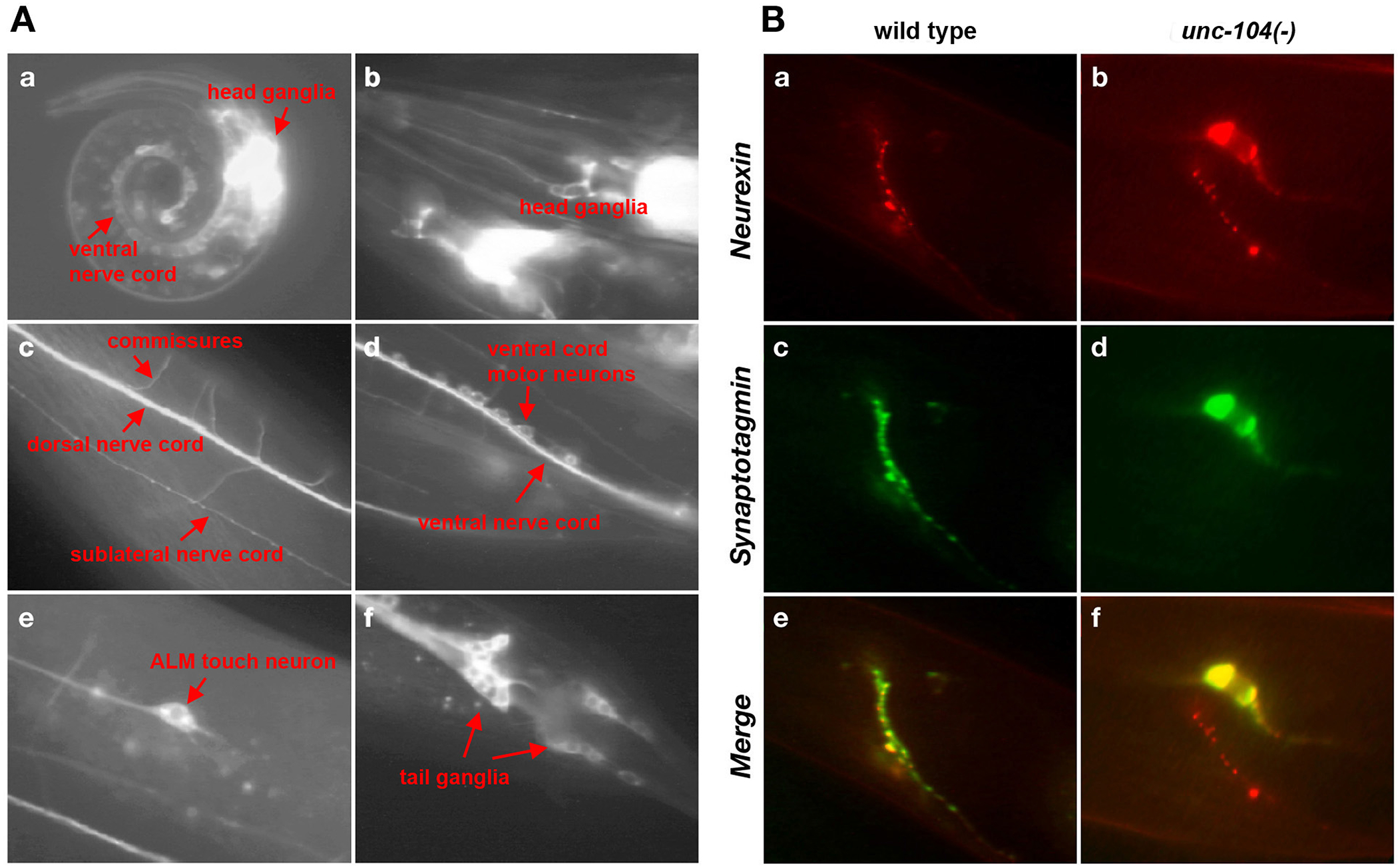
*nrx-1* expression and localization **A:** Expression of a *gfp*-tagged version of the genomic copy of the *nrx-1* locus (see Fig.1B for construct, expressed in transgenic animals. (a) Expression of both α– and β–neurexin isoforms using a *gfp* reporter construct shown in Fig.1B in a first larval stage animal. (b): Expression in the head region with pharynx and nerve ring. (c): Dorsal nerve cord and commissures. (d): Ventral nerve cord with motor and interneuron. (e): ALM neuron. (f): Neurons in tail ganglia. **B:** Synaptic localization of NRX-1 protein. (a) Localization of *gfp-*tagged *nrx-1*, fused to the promoter of the *sra-6* gene in the nerve ring of an adult wild type (wt) animal and in an *unc-104* mutant animal (b). (c) Localization of *bfp*-tagged *snt-1*, fused to the promoter of the *sra-6* gene in the nerve ring of an adult wild-type animal and in an *unc-104* mutant animal (d). (e,f) overlap of panels above.

The expression pattern of the *nrx-1* gene was determined by inserting *gfp* coding regions into a ~25kb genomic DNA piece that contains the entire *nrx-1* locus. The insertion point is shared by both isoforms and the reporter should therefore reveal the expression of both. We find that *nrx-1::gfp* is expressed broadly throughout the entire nervous system during all larval and adult stages (Fig.2A). Co-labeling with reporters that label subpopulation of individual neurons as well as the counting of neurons in several ganglia indicate that all neurons in the nervous system may express NRX-1. The only exception may be the canal-associated neuron CAN, a neuron which is known to make little if any synaptic contacts (White et al., 1986). No expression is observed in glia cells. The only cells outside the nervous system in which NRX-1::GFP could be observed are the spermathecal valve cells (data not shown).

NRX-1 localizes along the entire length of axonal projection (Fig.2); however, as synapses in *C.elegans* are made en passant within thick axonal bundles (nerve ring, nerve cord), the synaptic localization of a pan-neuronally expressed synaptic protein would be expected to result in a diffuse staining. To circumvent this problem, we set out to visualize NRX-1 in isolation, i.e. not neighbored by other axons. To this end, we expressed the α-isoform under control of the *sra-6* promoter which is expressed in 3 neurons with axonal projections into the nerve ring. Transgenic animals reveal that NRX-1α shows a synaptic localization pattern in the nerve ring (Fig.2B). Its localization overlaps with that of the presynaptic marker synaptotagmin/SNB-1 (Fig.2B), consistent with the presynaptic localization of neurexins in other systems (Sudhof, 2008). Unlike synaptic localization of synaptic vesicle proteins, such as synaptotagmin, this synaptic localization is independent of the kinesin protein UNC-104 (Fig.2B).

To examine sites of expression of the other neurexin family members, we generated *gfp* reporter gene fusions for *itx-1* and *nlr-1* by fusing 2977bp of sequences upstream of the *itx-1* predicted start site and 3526 bp upstream of the *nlr-1* predicted start site to *gfp* and generated transgenic animals expressing these constructs (*itx-1^prom^::gfp* and *nlr-1^prom^::gfp*).

**Fig.3:**
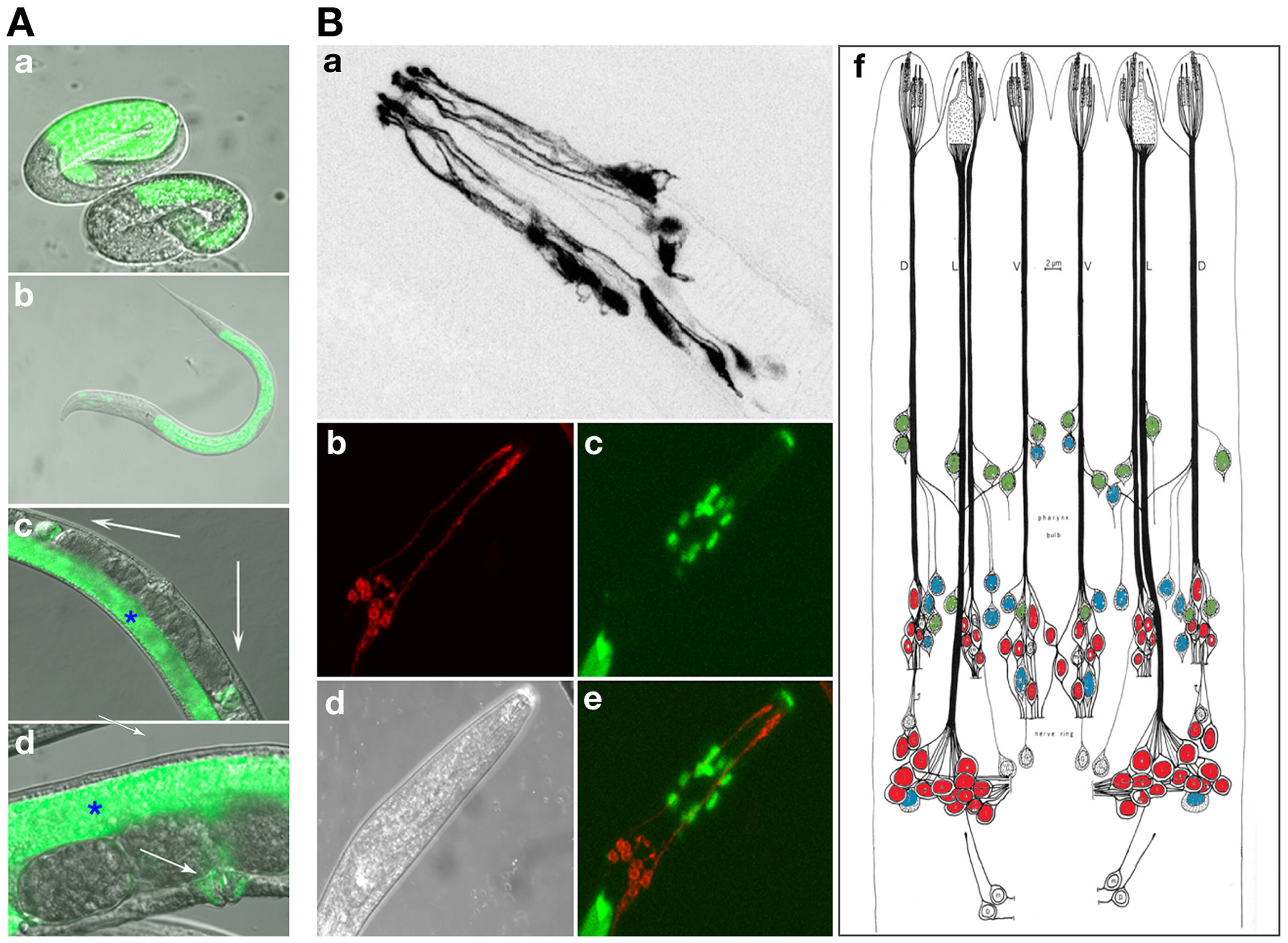
*itx-1* reporter gene expression **A:** *itx-1*^*prom*^*::gfp* expression in non-neuronal cells. (a) Intestinal expression in embryo and (b) in an L1 larva. (c,d) expression the excretory canal cell (panel D with Nomarksi), (e) expression in spermatheca (white arrows) and (f) in vulva (white arrows). Blue star in (e,f) indicates gut expression. **B:** *itx-1*^*prom*^*::gfp* expression in glial cells. (a) Expression in amphid socket cells; *gfp* signal is inverted for contrast; (b-e) same animal had shown with sensory neurons filled with dye, as previously described (Perkins et al., 1986). (b), socket cells with *itx-1*^*prom*^*::gfp* (c), Normarksi image (d) and overlap of red and green in (e). **C:** Sensory anatomy with associated sheath cell nuclei labeled in blue, socket cell nuclei in green and sensory neuron nuclei in red. Adapted from (Ward et al., 1975).

Expression of *itx-1^prom^::gfp* was observed in intestinal tract in embryos from 1.5 fold stage throughout post-embryonic larval development and adulthood (Fig.3). Furthermore, GFP could be detected in socket cells ensheathing the inner and outer labial neurons (Fig.3). Expression could also be observed in the spermatheca and vulva muscle cells (Fig.3). No other cell type display consistent reporter gene expression.

**Fig.4:**
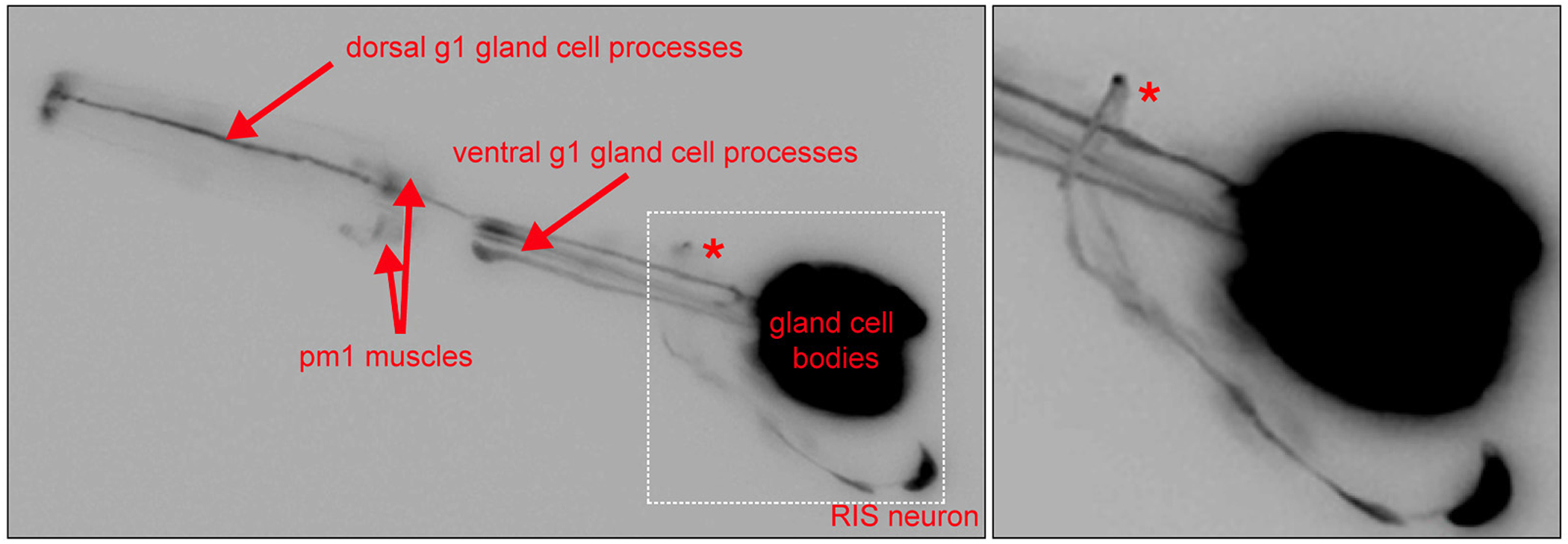
*nlr-1* reporter gene expression The head region of a transgenic young adult animal, expressing *nlr-1*^*prom*^*::gfp* is shown (see Fig.1B for structure of reporter gene). No consistent or strong *gfp* expression is observed elsewhere in the worm. Expression is the same in all postembryonic stages examined. Higher magnification of the boxed area is shown in the right panel.

Expression of *nlr-1^prom^::gfp* was observed in a pattern distinct from that of *itx-1^prom^::gfp*. Transgenic animals expressing *nlr-1^prom^::gfp* showed strong fluorescence in pharyngeal g1 and g2 gland cells, pharyngeal muscle cells and the unilateral, GABAergic RIS interneuron (Fig.4). This neuron is the only GABAergic neuron in *C. elegans* for which no function has been described yet (McIntire et al., 1993). These are the only cells in which strong and consistent expression is observed. Expression was observed during all stages of larval development and adulthood. We can not exclude the possibility the additional regulatory elements are located outside the region used for the reporter analysis. Yet the expression that we observe with this reporter represents a useful starting point for examining *nlr-1* function in these cells in the future.

### 2.3. ITX-1 protein is localized to the lateral membrane of gut epithelia and labels a unique membranous domain distinct from the apical junctions

**Fig.5:**
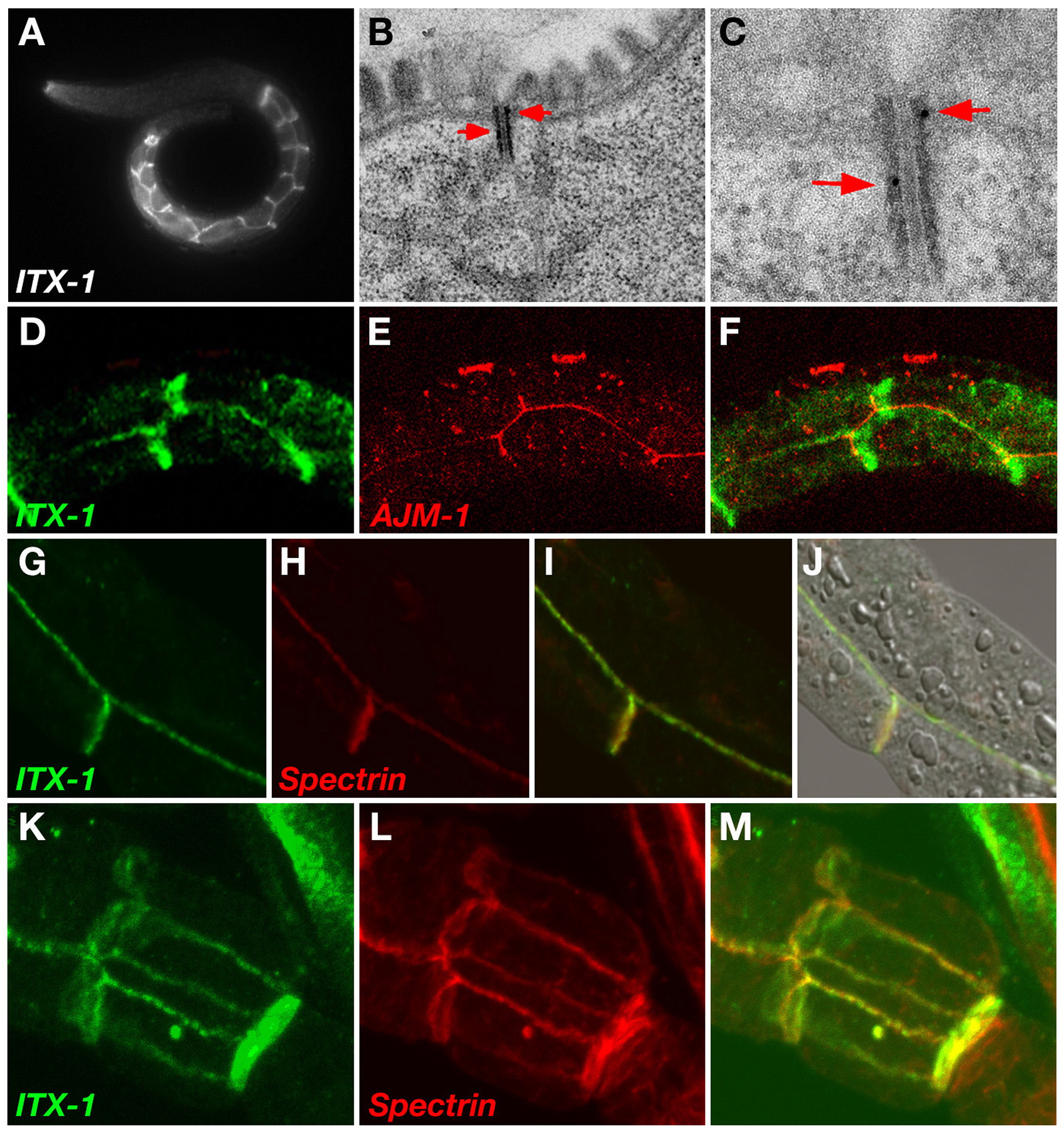
ITX-1 protein localization. **A:** ITX-1 localizes to adherence junctions in the intestine. anti-ITX-1 immunostaining of an L1 larval stage animal is shown. **B-C:** Immuno-EM with anti-ITX-1 antibodies. Arrows point to the electron dense gold particles of ITX-1 antibody that is situated in close vicinity to the typical *C. elegans* adherens junction and along the basolateral membrane of intestinal cells. **D-F:** ITX-1 antibody staining in the intestine, along the apical junction appears as a wider belt than the adherens junction marked using an antibody to AJM-1. **G-M:** Co-localization of ITX-1 and β-G-Spectrin in the intestine (G-J) and the pharynx (K-L).

We examined the subcellular localization of ITX-1 using polyclonal-antibodies raised against the 16 C-terminal amino acids of ITX-1. Affinity purified sera were used for staining whole mount N2 wild type worms of various developmental stages (Fig.5). Pre-immune sera and the flow-through fractions from the affinity-purification procedures were used as a negative control to ensure specificity of staining. Whole mount antibody staining established the expression of the protein at the baso-lateral membranes of gut epithelia in embryonic and post-embryonic stages. Its expression was restricted to gut and was not observed in other polarized epithelia, such as the pharynx or post-embryonic vulva, uterus, and hypodermis (Fig.5A,B). Immunoelectron microscopy experiments confirmed localization of ITX-1 to intestinal adherens junctions (Fig.5C). We could not detect embryonic or postembryonic ITX-1 antibody staining that we could unambiguously ascribe to sheath cells. It is possible that ITX-1 is only transiently expressed in these cells while they develop during embryogenesis.

To characterize the specific membrane domain occupied by ITX-1 in the intestine, we conducted co-localization studies with various epithelial membrane markers. As indicated in Fig.5C, ITX-1 defines a lateral membrane domain basal to the apical junction, a domain containing the adhesion proteins AJM-1 and DLG-1 (Lynch and Hardin, 2009). However, ITX-1 staining along the apical junction appears as a wider belt differently than the typical AJM/DLG pattern. Moreover, ITX-1 localization is independent of AJM-1, as *ajm-1* null mutant embryos show normal ITX-1 staining (data not shown). Co-labeling of ITX-1 with anti β-G Spectrin show that it co-localizes with ITX-1 along the entire lateral membrane of gut epithelia (Fig.5C). However, like the previously reported RNAi-mediated depletion of β-G Spectrin (Hammarlund et al., 2000; Moorthy et al., 2000), RNAi depletion of *itx-1* does not result in the disruption of gut morphology (data not shown). It is possible that the function of these two components is possibly masked by a redundant role conferred by other lateral membrane and membrane associated components.

## 3. DISCUSSION

The *C. elegans* genome encodes 3 core members of the neurexin superfamily and one more divergent member. Well characterized interaction partners of members of the vertebrate neurexin superfamily are also conserved in *C. elegans*. The most prominent vertebrate neurexin binding partner, the neuroligin protein, is conserved in *C. elegans* and called NRG-1 (Feinberg et al., 2008; Hunter et al., 2010). Moreover, there is a single ortholog of the vertebrate CASPR-binding protein contactin, called *rig-6* in *C. elegans* (Schwarz et al., 2009).

Bearing the caveats of reporter gene analysis in mind, we find that members of the neurexin-superfamily of proteins show diverse expression patterns within and outside the nervous system. Pursuing the expression and localization of two of the neurexin superfamily members in more detail we find that NRX-1 is synaptically localized, while ITX-1, a CASPR-ortholog localizes to the baso-lateral membrane domain of intestinal epithelia.

The canonical binding partner of neurexins, neuroligin, is expressed in pre- and postsynaptic specialization of *C. elegans* (Feinberg et al., 2008; Hunter et al., 2010). At this point, we can not exclude that neurexin is also not only pre- but also postsynaptically localized. Yet in any case, both neuroligin and neurexin appear to be synaptically localized proteins, as are their vertebrate counterparts (Lise and El-Husseini, 2006; Sudhof, 2008).

CASPR binds to the IgSF member contactin in a *cis*-configuration (Peles et al., 1997). The *C. elegans* contactin-encoding gene *rig-6* is expressed in a restricted number of neurons and several cell types outside the nervous system (Schwarz et al., 2009). There is little overlap in the expression of *rig-6* with *nlr-1* and *itx-1*. More extensive overlaps may be observed once the expression pattern of *rig-6* and *nlr-1* is more definitely studied by antibody staining, rather than with reporter genes, which may be lacking relevant regulatory regions.

Our findings on ITX-1 localization reveal an additional layer of complexity in the structure of apical membrane domains. Recent studies have indicated that these junctions are composed of at least three sub-domains each with a unique molecular signature: A subapical domain contains the transmembrane protein Crumbs-1 (CRB-1) and NFB-2 (an intermediate component), the *C. elegans* cadherin, α/β catenin HMP-1/HMR-1/HMR-2 system occupy an adjacent domain and the basal unit comprises DLG-1 and AJM-1 (Lynch and Hardin, 2009). Based on genetic interaction and RNAi knockdown experiments it was demonstrated that adhesion between adjacent intestinal cells, apical junction assembly, tissue integrity and tubulogenesis of gut lumen is dependent on redundant or parallel systems that operate in different regions of the plasma membrane and appear to be under different control mechanisms. In the wildtype embryo, the AJM-1/DLG-1 complex is predominantly under the control of basolaterally expressed LET-413 (Legouis et al., 2000; McMahon et al., 2001). In contrast, the establishment of the catenin–cadherin complex is barely affected in a *let-413* or *dlg-1* background (Bossinger et al., 2004; Legouis et al., 2000; McMahon et al., 2001). This points to the existence of additional control mechanisms operating from different membrane domains. Consistent with this notion, we find that ITX-1 localization does not depend on AJM-1. Therefore, despite its simple appearance, the apical junction is assembled and maintained by orchestrated hierarchies of various molecular determinants in which ITX-1 plays an as yet unknown role.

Intriguingly, other proteins that were previously shown to be expressed in or close to this domain, such as AJM-1 localize to various polarized membrane domains besides the intestine (e.g. pharynx or epidermis). In contrast, ITX-1 only localizes to this membrane domain in the gut. This finding demonstrates that the composition of junctional complexes is distinct in different cell types and suggests that the intestine may have special needs for building such structures and/or for maintaining its structural integrity.

In conclusion, our expression studies provide a starting point for a functional analysis of members of the neurexin superfamily, making use of the specific tools available in *C. elegans*.

## 4. EXPERIMENTAL PROCEDURES

### 4.1. GFP expression constructs

2977 bp upstream to the ATG of *itx-1(W03D8.6)* were amplified with forward primer 5’-CGGTAACACTGCAGAGTAAATTG-3’ (containing a PstI site) and. reverse primer 5’-GGGGCCCGGGTGAAATAGAGAGC-3’ (containing a XmaI site), digested with PstI/XmaI and subcloned into the multiple cloning site of pPD.95.75 *C. elegans* expression vector (Fire lab 1999 vector Supplement kit). This construct was designated pPD95.75-itxpr. 3526 bp of the promoter sequence located upstream to the ATG of *nlr-1* coding region were amplified with forward primer 5’- AAAGGCTTGCTGATAGAAGTATAACTG -3’ and reverse primer 5’-CATTCGGATCCGGAGGGCATATGTGTGTGG-3’ and also cloned into the pPD.95.75 vector. The construct was designated pPD95.75-nlrprB. The *nrx-1::gfp* expression construct was generated by homologous recombination in bacteria (Soutschek, 2000). Trangenic animals were generated using *rol-6* as injection marker.

### 4.2. *C. elegans* antibodies and antibody staining

Antibodies were raised against ITX-1 protein by immunizing mice and rabbits with a peptide based hapten-carrier conjugate. The C-terminus peptide sequence KYLHDEDIPLHMAPTI (A2) consisting of amino acids 1483-1497 of the ITX-1 protein was used. Affinity purified antibodies were used in 1:200 dilution for whole mount immuno-staining, and 1:1000 for IP and western blots (the Western Blot showed a band of the expected size; data not shown). Pre-immune sera and the flow through fractions from the affinity-purification procedures were used as negative controls to ensure specificity of staining.

The monoclonal antibodies MH27 that recognizes junctional adhesion molecule-1 (AJM-1)(Francis and Waterston, 1991) and MH33 that recognizes intermediate filament protein IFB-2 (Francis and Waterston, 1985) were used at a dilution of 1:1000 of ascetic fluid from mice immunized with the respective hybridoma cell line. Rabbit polyclonal anti β-G Spectrin was kindly provided by Vann Bennet (Moorthy et al., 2000).

Larvae and adult worms were stained according to the standard Finney-Ruvkun whole mount protocol (Finney and Ruvkun, 1990). Embryos immunostaining followed the freeze-cracking fixation protocol (Miller and Shakes, 1995). Primary antibodies were used in the following dilutions: rabbit and mouse αITX-1 1:200, mAb NH33 and MH27 at 1:1000 and rabbit αβ-G Spectrin at 1:250. Immunofluorescence acquisition and documentation were done with a BioRad confocal microscope.

### 4.3. Immunoelectron microscopy

Mix stage population of N2 worms were fixed and embedded for electron microscopy using High pressure freezing (HPF) followed by freeze substitution at gradual temperature increase. Samples were kept for 3 days at −90°C in anhydrous acetone containing 0.2% tannic acid and then warmed to −30°C during 30 hours. Samples were then consecutively washed with 100% acetone, 100% ethanol and then incubated for 1 hour in ethanol containing 0.5% Uranyl acetate. Samples were then embedded in Lowycril K4M, and polymerized at −30°C. The hardened samples were brought to room temperature and sectioned using a microtome (Leica EM Ultracut, Vienna, Austria) equipped with a diamond knife (Diatome AG, Biel Switzerland) and place on nickel grids. The sections were incubated with αITX-1 antibody and then with α rabbit IgG secondary antibody conjugated to gold particles and double stained with uranyl acetate/lead citrate. Samples were observed in a FEI Tecnai T12 transmission electron microscope.

## References

[R1] Bhat MA, Rios JC, Lu Y, Garcia-Fresco GP, Ching W, St Martin M, Li J, Einheber S, Chesler M, Rosenbluth J, Salzer JL and Bellen HJ (2001) Axon-glia interactions and the domain organization of myelinated axons requires neurexin IV/Caspr/Paranodin. Neuron 30, 369–83. 11395000 10.1016/s0896-6273(01)00294-x

[R2] Bossinger O, Fukushige T, Claeys M, Borgonie G and McGhee JD (2004) The apical disposition of the Caenorhabditis elegans intestinal terminal web is maintained by LET-413. Dev Biol 268, 448–56. 15063180 10.1016/j.ydbio.2004.01.003

[R3] Boyle ME, Berglund EO, Murai KK, Weber L, Peles E and Ranscht B (2001) Contactin orchestrates assembly of the septate-like junctions at the paranode in myelinated peripheral nerve. Neuron 30, 385–97. 11395001 10.1016/s0896-6273(01)00296-3

[R4] Colavita A and Tessier-Lavigne M (2003) A Neurexin-related protein, BAM-2, terminates axonal branches in C. elegans. Science 302, 293–6. 14551437 10.1126/science.1089163

[R5] Feinberg EH, Vanhoven MK, Bendesky A, Wang G, Fetter RD, Shen K and Bargmann CI (2008) GFP Reconstitution Across Synaptic Partners (GRASP) defines cell contacts and synapses in living nervous systems. Neuron 57, 353–63. 18255029 10.1016/j.neuron.2007.11.030

[R6] Feinberg K, Eshed-Eisenbach Y, Frechter S, Amor V, Salomon D, Sabanay H, Dupree JL, Grumet M, Brophy PJ, Shrager P and Peles E (2010) A glial signal consisting of gliomedin and NrCAM clusters axonal Na+ channels during the formation of nodes of Ranvier. Neuron 65, 490–502. 20188654 10.1016/j.neuron.2010.02.004 PMC2831809

[R7] Finney M and Ruvkun G (1990) The unc-86 gene product couples cell lineage and cell identity in C. elegans. Cell 63, 895–905. 2257628 10.1016/0092-8674(90)90493-x

[R8] Francis GR and Waterston RH (1985) Muscle organization in Caenorhabditis elegans: localization of proteins implicated in thin filament attachment and I-band organization. J Cell Biol 101, 1532–49. 2413045 10.1083/jcb.101.4.1532 PMC2113919

[R9] Francis R and Waterston RH (1991) Muscle cell attachment in Caenorhabditis elegans. J Cell Biol 114, 465–79. 1860880 10.1083/jcb.114.3.465 PMC2289102

[R10] Gollan L, Salomon D, Salzer JL and Peles E (2003) Caspr regulates the processing of contactin and inhibits its binding to neurofascin. J Cell Biol 163, 1213–8. 14676309 10.1083/jcb.200309147 PMC2173730

[R11] Hammarlund M, Davis WS and Jorgensen EM (2000) Mutations in beta-spectrin disrupt axon outgrowth and sarcomere structure. J Cell Biol 149, 931–42. 10811832 10.1083/jcb.149.4.931 PMC2174563

[R12] Hunter JW, Mullen GP, McManus JR, Heatherly JM, Duke A and Rand JB (2010) Neuroligin-deficient mutants of C. elegans have sensory processing deficits and are hypersensitive to oxidative stress and mercury toxicity. Dis Model Mech 3, 366–76. 20083577 10.1242/dmm.003442 PMC4068633

[R13] Legouis R, Gansmuller A, Sookhareea S, Bosher JM, Baillie DL and Labouesse M (2000) LET-413 is a basolateral protein required for the assembly of adherens junctions in Caenorhabditis elegans. Nat Cell Biol 2, 415–22. 10878806 10.1038/35017046

[R14] Lise MF and El-Husseini A (2006) The neuroligin and neurexin families: from structure to function at the synapse. Cell Mol Life Sci 63, 1833–49. 16794786 10.1007/s00018-006-6061-3 PMC11136152

[R15] Lynch AM and Hardin J (2009) The assembly and maintenance of epithelial junctions in C. elegans. Front Biosci 14, 1414–32. 10.2741/3316 PMC2896272 19273138

[R16] McIntire SL, Jorgensen E, Kaplan J and Horvitz HR (1993) The GABAergic nervous system of Caenorhabditis elegans. Nature 364, 337–41. 8332191 10.1038/364337a0

[R17] McMahon L, Legouis R, Vonesch JL and Labouesse M (2001) Assembly of C. elegans apical junctions involves positioning and compaction by LET-413 and protein aggregation by the MAGUK protein DLG-1. J Cell Sci 114, 2265–77. 11493666 10.1242/jcs.114.12.2265

[R18] Miller DM 3rd and Shakes D (1995) Immunofluorescence Microscopy. In Epstein HF and Shakes D (eds.), Caenorhabditis elegans: Modern Biological Analysis of an organism, Academic Press, Vol. 48, pp. 365–395.

[R19] Missler M, Fernandez-Chacon R and Sudhof TC (1998) The making of neurexins. J Neurochem 71, 1339–47. 9751164 10.1046/j.1471-4159.1998.71041339.x

[R20] Moorthy S, Chen L and Bennett V (2000) Caenorhabditis elegans beta-G spectrin is dispensable for establishment of epithelial polarity, but essential for muscular and neuronal function. J Cell Biol 149, 915–30. 10811831 10.1083/jcb.149.4.915 PMC2174577

[R21] Peles E, Joho K, Plowman GD and Schlessinger J (1997) Close similarity between Drosophila neurexin IV and mammalian Caspr protein suggests a conserved mechanism for cellular interactions. Cell 88, 745–6. 9118217 10.1016/s0092-8674(00)81920-0

[R22] Peles E and Salzer JL (2000) Molecular domains of myelinated axons. Curr Opin Neurobiol 10, 558–65. 11084317 10.1016/s0959-4388(00)00122-7

[R23] Perkins LA, Hedgecock EM, Thomson JN and Culotti JG (1986) Mutant sensory cilia in the nematode Caenorhabditis elegans. Dev Biol 117, 456–87. 2428682 10.1016/0012-1606(86)90314-3

[R24] Poliak S and Peles E (2003) The local differentiation of myelinated axons at nodes of Ranvier. Nat Rev Neurosci 4, 968–80. 14682359 10.1038/nrn1253

[R25] Poliak S, Salomon D, Elhanany H, Sabanay H, Kiernan B, Pevny L, Stewart CL, Xu X, Chiu SY, Shrager P, Furley AJ and Peles E (2003) Juxtaparanodal clustering of Shaker-like K+ channels in myelinated axons depends on Caspr2 and TAG-1. J Cell Biol 162, 1149–60. 12963709 10.1083/jcb.200305018 PMC2172860

[R26] Quevillon E, Silventoinen V, Pillai S, Harte N, Mulder N, Apweiler R and Lopez R (2005) InterProScan: protein domains identifier. Nucleic Acids Res 33, W116–20. 15980438 10.1093/nar/gki442 PMC1160203

[R27] Schwarz V, Pan J, Voltmer-Irsch S and Hutter H (2009) IgCAMs redundantly control axon navigation in Caenorhabditis elegans. Neural Dev 4, 13. 19341471 10.1186/1749-8104-4-13 PMC2672934

[R28] Soutschek J (2000) Die Rolle von Neurexin-1 in der Entwicklung des Nervensystems von C. elegans. Eberhard-Karls-Universität Tübingen, pp. 123.

[R29] Sudhof TC (2008) Neuroligins and neurexins link synaptic function to cognitive disease. Nature 455, 903–11. 18923512 10.1038/nature07456 PMC2673233

[R30] Tabuchi K and Sudhof TC (2002) Structure and evolution of neurexin genes: insight into the mechanism of alternative splicing. Genomics 79, 849–59. 12036300 10.1006/geno.2002.6780

[R31] Traka M, Goutebroze L, Denisenko N, Bessa M, Nifli A, Havaki S, Iwakura Y, Fukamauchi F, Watanabe K, Soliven B, Girault JA and Karagogeos D (2003) Association of TAG-1 with Caspr2 is essential for the molecular organization of juxtaparanodal regions of myelinated fibers. J Cell Biol 162, 1161–72. 12975355 10.1083/jcb.200305078 PMC2172849

[R32] Ullrich B, Ushkaryov YA and Sudhof TC (1995) Cartography of neurexins: more than 1000 isoforms generated by alternative splicing and expressed in distinct subsets of neurons. Neuron 14, 497–507. 7695896 10.1016/0896-6273(95)90306-2

[R33] Ushkaryov YA, Petrenko AG, Geppert M and Sudhof TC (1992) Neurexins: synaptic cell surface proteins related to the alpha-latrotoxin receptor and laminin. Science 257, 50–6. 1621094 10.1126/science.1621094

[R34] Ward S, Thomson N, White JG and Brenner S (1975) Electron microscopical reconstruction of the anterior sensory anatomy of the nematode Caenorhabditis elegans.?2UU. J Comp Neurol 160, 313–37. 1112927 10.1002/cne.901600305

[R35] White JG, Southgate E, Thomson JN and Brenner S (1986) The structure of the nervous system of the nematode *Caenorhabditis elegans*. Philosophical Transactions of the Royal Society of London B. Biological Sciences 314, 1–340. 22462104 10.1098/rstb.1986.0056

